# Flexible design in the stomatopod dactyl club

**DOI:** 10.1107/S2052252523002075

**Published:** 2023-03-14

**Authors:** Thorbjørn Erik Køppen Christensen, Jia Qing Isaiah Chua, Nina Kølln Wittig, Mads Ry Vogel Jørgensen, Innokenty Kantor, Jesper Skovhus Thomsen, Ali Miserez, Henrik Birkedal

**Affiliations:** a Center for Integrated Materials Research (iMAT), Department of Chemistry and iNANO, Aarhus University, 14 Gustav Wieds Vej, Aarhus C. 8000, Denmark; b Sino-Danish College (SDC), University of Chinese Academy of Sciences, Aarhus University, Aarhus C., Denmark; c Biological and Biomimetic Materials Laboratory, Center for Sustainable Materials (SusMat), School of Materials Science and Engineering, Nanyang Technological University, 50 Nanyang Avenue, 639798, Singapore; d DanMAX, MAX IV laboritory, Fotongatan 2, Lund, Sweden; e Department of Physics, Danish Technological University, Fysikvej 310, Lyngby 2800, Denmark; f Department of Biomedicine, Aarhus University, Wilhelm Meyers Allé 3, Aarhus C. 8000, Denmark; ESRF, France

**Keywords:** biomineralization, stomatopods, crystal orientation, composite materials, polymorphism

## Abstract

The stomatopod is a fascinating animal that uses its weaponized appendage dactyl clubs for breaking mollusc shells. Dactyl clubs are a well studied example of biomineralized hierarchical structures. Most research has focused on the regions close to the action, namely the impact region and surface composed of chitin and apatite crystallites. Further away from the site of impact, the club has lower mineralization and more amorphous phases; these areas have not been as actively studied as their highly mineralized counterparts. This work focuses on the side of the club, in what is known as the periodic and striated regions. A combination of laboratory micro-computed tomography, synchrotron X-ray diffraction mapping and synchrotron X-ray fluorescence mapping has shown that the mineral in this region undergoes the transition from an amorphous to a crystalline phase in some, but not all, clubs. This means that this side region can be mineralized by either an amorphous phase, calcite crystallites or a mixture of both. It was found that when larger calcite crystallites form, they are organized (textured) with respect to the chitin present in this biocomposite. This suggests that chitin may serve as a template for crystallization when the side of the club is fully mineralized. Further, calcite crystallites were found to form as early as 1 week after moulting of the club. This suggests that the side of the club is designed with a significant safety margin that allows for a variety of phases, *i.e.* the club can function independently of whether the side region has a crystalline or amorphous mineral phase.

## Abstract 2

The stomatopod dactyl club has a complex structure featuring an impact region, periodic regions and striated regions. The side region of the club contains both periodic and striated parts. The side region is considered to be mineralized by inorganic amorphous phases. This work shows that these phases crystallize to calcite, generating a macroporous structure early in the biomineralization process. This phase transition is common, but does not occur in every club. Thus, the appearance of the macroporous calcite structure must reflect a natural flexible design allowing for structural plasticity, such that the club remains functionally viable even upon large changes in the mineral phase microstructure.

## 1. Introduction

**Figure 1:**
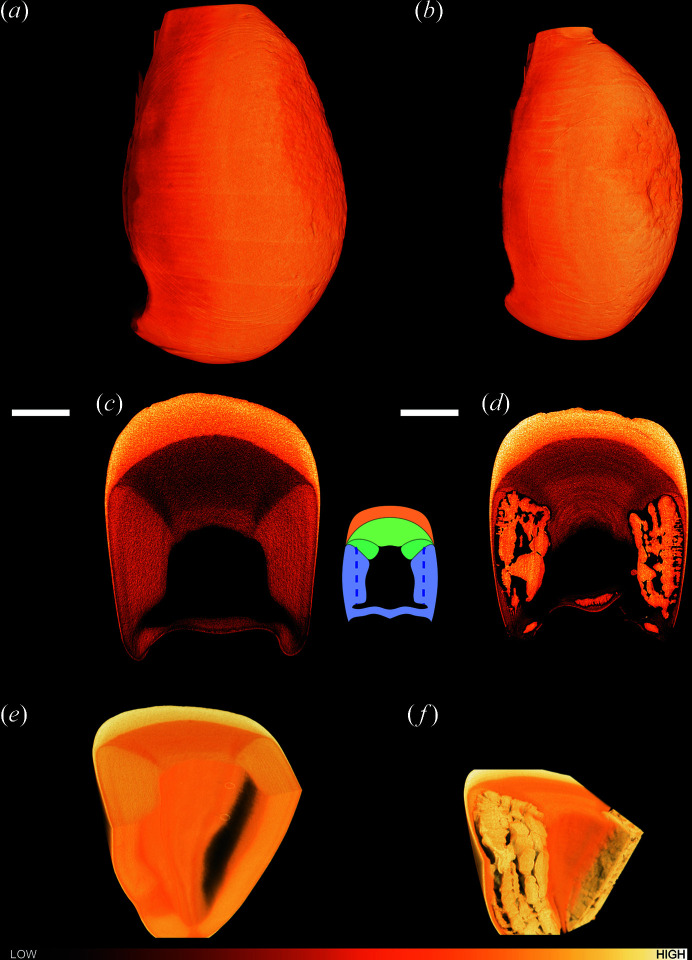
Lab-µCT of clubs (*a*), (*c*), (*e*) 1 and (*b*), (*d*), (*f*) 2, with (*a*), (*b*) 3D renderings; (*c*), (*d*) a virtual slice through the clubs; and (*e*), (*f*) cut-out 3D renderings of the lower front half of the clubs shown from the back of the clubs. For visibility, the orientation of the cut-out 3D renderings (*e*) and (*f*) differs from those of the full club 3D renderings (*a*) and (*b*). Scale bars in (*c*) and (*d*) are 1 mm. The inset between (*c*) and (*d*) shows a sketch of the different regions in the club with the impact region in orange, the periodic region in green, and the striated and side periodic regions in blue; this was inspired by the sketch by Weaver *et al.* (2012 ▸). The colour gradient bar illustrates the material density.

The stomatopod *Odontodactylus scyllarus* uses its two dactyl clubs to destroy its prey with bullet-like acceleration (Patek *et al.*, 2004 ▸). Given the extreme biomechanics involved in stomatopod hunting, the club material has garnered much interest (Dong *et al.*, 2022 ▸; Chua *et al.*, 2023 ▸, 2021 ▸; Amini *et al.*, 2019 ▸; Zhang *et al.*, 2016 ▸; Weaver *et al.*, 2012 ▸; Patek *et al.*, 2004 ▸; Currey *et al.*, 1982 ▸; Caldwell & Dingle, 1976 ▸). More recent studies have focused on the micro- and nanostructure of the dactyl club. Weaver *et al.* (2012 ▸) defined three major structural areas in the club (Fig. 1 ▸): (1) the impact region, indicated in orange in the inset of Fig. 1 ▸, is highly mineralized with a mixture of highly substituted fluoro- and hydroxyl-apatite (Ap) crystals organized with semicrystalline chitin in a plywood motif; (2) the periodic region, which contains laminated chitin in a helical structure surrounded by amorphous calcium phosphate (ACP) and amorphous calcium carbonate (ACC); and (3) the striated region that contains a mixture of ACC and ACP interwoven with parallel chitin fibres. The periodic region is located both beneath the impact region (green in the Fig. 1 ▸ inset) and in the side of the club near the cavity (blue, left of the vertical dashed line in the inset), whereas the striated region is situated in the side of the club near its outer edge (blue, right of the vertical dashed line in the inset). Herein, we refer to the striated region and the part of the periodic region in the side area of the club as the side region; this region is shown in blue in the inset in Fig. 1 ▸.

After Weaver *et al.* (2012 ▸) defined the club morphology, the club biomineralization process was studied by following club development as a function of time since moulting (Amini *et al.*, 2019 ▸). The club mineralized from the outside in, with the impact region forming first (Amini *et al.*, 2019 ▸). The nanostructure of the impact region was shown to be critical in absorbing impact by enabling quasi-plastic deformation at contact points (Amini *et al.*, 2015 ▸). Furthermore, fracture mechanics studies showed that this region exhibits an *R*-curve-type behaviour, thus enhancing the overall fracture tolerance (Dong *et al.*, 2022 ▸; Chua *et al.*, 2021 ▸). The focus of these preceding studies was mainly the impact region or the impact surface (Grunenfelder *et al.*, 2018 ▸; Weaver *et al.*, 2012 ▸). Both the impact region and impact surface are of great interest, but alone these regions do not describe the stomatopod dactyl club structure in full. In the studies that investigated the periodic and striated regions, they were not explored to the same degree as the impact region. Therefore, the aim of the present study was to investigate the structure of the striated part of the side of the club and lateral periodic regions situated in the side of the club using X-ray diffraction (XRD), X-ray fluorescence (XRF) and laboratory micro-computed tomography (lab-µCT). Through these investigations we established that the side of the club in some cases does crystallize, but not to Ap – as one might expect from it being the dominant crystal in the impact region – but rather to calcite. We observe that this crystallization can happen already during the first week after moulting. The fact that the lateral periodic region can crystallize while the club is still functional suggests a high degree of flexibility in its design. Therefore, we speculate that this flexibility may reflect a design safety margin. In total, seven clubs were investigated, some using lab-µCT, some using XRD and XRF mapping, and some using lab-µCT as well as XRD and XRF scanning, this is summarized in Table 1 ▸.

## 2. Methods and materials

### 2.1. Sample preperation

Stomatopods of the species *Odontodactylus scyllarus* were acquired from aquarist vendors in Singapore and Stockholm. The animals obtained from Singapore were kept in aquaria with artificial seawater in the same manner as in our previous work (Chua *et al.*, 2023 ▸; Amini *et al.*, 2019 ▸). From the live animals, three mature clubs were collected when shed during moulting (clubs 1, 2 and 6), and another club was collected one week after moulting (club 7). The animals obtained from the aquarist vendor in Stockholm were sacrificed on arrival in Aarhus, stored in 50% ethanol for one day, followed by 70% ethanol, and their right clubs (clubs 3–5) were subsequently isolated from the ethanol-preserved specimens.

For synchrotron radiation experiments, the clubs were first embedded in epoxy resin (EpoFix, Struers, Ballerup, Denmark), after which, axial sections were cut with a low-speed saw using a diamond cut-off wheel (accutom-5, with M1D15, Struers, Ballerup, Denmark). Then the slices were polished with SiC abrasive paper (Struers, Ballerup, Denmark) to a thickness below 100 µm.

### 2.2. Laboratory computed tomography

Lab-µCT was conducted using the Aarhus X-ray imaging alliance (AXIA) infrastructure (Wittig *et al.*, 2022 ▸). Clubs 1 and 2 were scanned in a Scanco µCT35 (Scanco Medical, Brüttisellen, Switzerland) setup mounted on a plastic sample holder. The clubs were scanned using an energy of 55 kVp, an isotropic voxel size of 3.5 µm and an integration time of 2.4 s.

Three whole animals were scanned in full in an Xradia 620 Versa (Zeiss, Oberkochen, Germany). The smallest animal (animal 3, Table 1 ▸) was mounted in a falcon tube and the two larger animals (4 and 5) were mounted in custom-made glass cylinders. All animals were suspended in a mixture of 70% ethanol and ethanolized agar gel cubes for stabilization (Walsh *et al.*, 2021 ▸). The smaller animal (animal 3) was scanned at 80 kVp with an isotropic voxel size of 22 µm, and the larger animals were scanned at 140 kVp with an isotropic voxel size of 42 µm; all animals were scanned with an exposure of 1 s per frame. The scans of the entire animals had restrictions on both the spatial and the grey-scale resolution, as both the exoskeleton and the club needed to be visible, and the stomatopod needed to fit in the field of view within the jars containing them. Thus, to better resolve the features of the clubs, the right clubs were rescanned after extraction. The right clubs (clubs 3–5) of the animals were extracted and embedded in EpoFix (Struers, Ballerup, Denmark) before being scanned with an isotropic voxel size of 4.5 µm, an energy of 80 kVp and exposure times ranging from 2.5 to 35 s depending on the size of the embedded clubs.

### 2.3. Synchrotron X-ray experiments

XRF and XRD experiments were carried out at the hard X-ray micro/nanoprobe P06 beamline at PETRA-III at the Deutsche Elektronen-Synchrotron (DESY, Hamburg, Germany), and XRD experiments were also carried out at the DanMAX beamline at MAXIV (MAXIV, Lund, Sweden).

Sections of clubs 6 and 7 were mounted between two layers of Ultralene foils, and the samples were scanned at P06 using a pencil beam focused to 500 × 380 nm (H × V) at an energy of 16.5 keV. The samples were scanned by continuous scans with a step size of 10 × 10 µm with 10 ms exposure for overview scans and a step size of 500 × 500 nm and 100 ms exposure for high-resolution scans. XRD data were collected using an EigerX 4M detector (Dectris, Baden-Daettwil, Switzerland) with a sample-to-detector distance of 141.1 mm, and XRF data was collected using a MAIA 384C detector (Ryan *et al.*, 2010 ▸). No background was subtracted from the XRD data.

Slices from clubs 3–5 were mounted on Kapton tape and scanned at DanMAX with a pencil beam focused to 20 × 10 µm (H × V) at an energy of 35 keV and a step size of 10 × 10 µm and 249 ms exposure per point. XRD data were collected using a Pilatus3X 2MCdTe detector (Dectris, Baden-Daettwil, Switzerland) with a sample-to-detector distance of 518 mm. One slice of club 3 (*see infra*) was then heat-treated at 373 K in a vacuum oven (MTI Corporation, Richmond CA, USA) for 2.5 h to simulate drying, and rescanned with an exposure time of 44 ms per point. A Kapton background signal was measured using an exposure time of 1 s and subsequently scaled and subtracted from all diffractograms.

### 2.4. Data treatment

The XRD data were integrated using the *MatFRAIA* algorithm (Jensen *et al.*, 2022 ▸) implemented at several beamlines at MAX IV including NanoMAX (Bjorling *et al.*, 2021 ▸) and DanMAX (Jensen *et al.*, 2022 ▸). This resulted in 1D diffraction patterns usable for phase composition analysis. The P06 data were also integrated to azimuthally resolved diffraction data (360 azimuthal angular bins) for orientation analysis. The orientations of mineral crystallites and chitin were analysed using the angular Gaussian fitting (AGUF) method (Jensen *et al.*, 2022 ▸) that fits the azimuthal intensity distribution of a Bragg peak in a variation of the methods previously used for small-angle X-ray scattering (SAXS) data (Törnquist *et al.*, 2020 ▸; Bünger *et al.*, 2010 ▸; Rinnerthaler *et al.*, 1999 ▸; Fratzl *et al.*, 1996 ▸). The AGUF analysis was performed on the calcite (104) and (110) peaks as well as the chitin (013) peak. For phase identification, a model calcite diffraction pattern was calculated at 16.5 keV from literature data (Maslen *et al.*, 1995 ▸) using *Mercury* (Macrae *et al.*, 2020 ▸). For determination of the spatial distribution of phases from the azimuthally integrated (1D) diffractograms, the Ap (002) peak; the chitin (110) and (013) peaks; and the calcite (104), (110), and (202) peaks were used. The XRF data were analysed using the *Geopixe* software (Ryan *et al.*, 2010 ▸, 1990 ▸) to obtain Ca content maps of clubs 6 and 7. The lab-µCT data were visualized using *Dragonfly* (ORS, Montréal, Canada) for clubs 1, 2 and 3–5.

**Figure 2:**
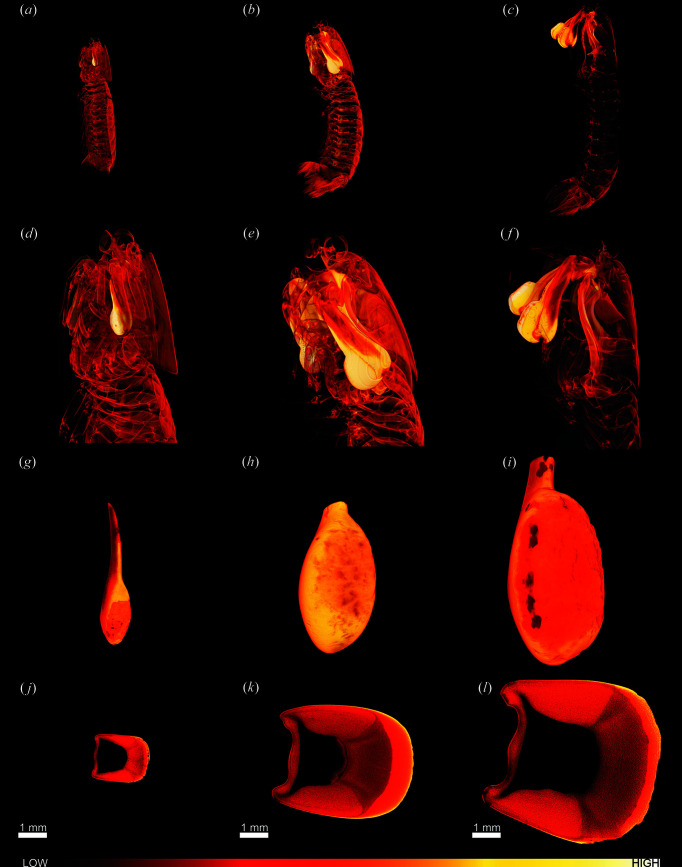
Lab-µCT of animals 3–5 and clubs 3–5. (*a*)–(*c*) Rendering of full animals. (*d*)–(*f*) Digital zoom-in on the heads of the animals from the data in (*a*)–(*c*). (*g*)–(*i*) Renderings of clubs 3–5 from higher-resolution measurements on extracted right clubs from the animals in panels (*a*)–(*c*). (*j*)–(*k*) Virtual slices through clubs 3–5. The lengths of the animals were (*a*), (*d*), (*g*), (*j*) 11 cm; (*b*), (*e*), (*h*), (*k*) 13 cm; and (*c*), (*f*), (*i*), (*l*) 16 cm long. The colour gradient bar illustrates the material density.

The XRD data were collected in a raster scanning mode. This made it possible to carry out a positionally resolved orientation analysis on the two regions scanned. This was performed on a peak-by-peak basis using the AGUF method (Jensen *et al.*, 2022 ▸) that fits the azimuthal intensity distribution. We note that a different technique for orientation analysis, also based on the azimuthal scattering angle, has previously been used on the tail armour of the stomatopod (Zhang *et al.*, 2016 ▸); this method is visualized in full 3D, which is not possible for scans of the size used in the present work. For calcite, the peaks used for orientation determination were the (104) and (110) peaks, which together cover crystallites in all orientations [Fig. 2 ▸(*c*)]. For chitin, the (013) peak was used for orientational analysis, whereas the (110) peak was used only for spatial localization of chitin as it had no observable in-plane texture [Fig. 2 ▸(*c*)].

## 3. Results and discussion

We first discuss the club microstructure obtained from lab-µCT measurements and then the results of the analysis of the synchrotron XRD and XRF data.

### 3.1. Lab-µCT experiments

The surfaces of the impact regions of clubs 1 and 2 rendered with laboratory µCT reveal that both clubs are roughened due to wear [Figs. 1 ▸(*a*) and 1(*b*)]. Interestingly, virtual axial cross-sections through the centre of the clubs showed remarkable morphological differences between the two clubs [Figs. 1 ▸(*c*) and 1 ▸(*d*)]. In club 1, the structure was similar to that expected from previous research (Weaver *et al.*, 2012 ▸) with a streamlined uniform morphology. Instead, we sometimes observed larger morphological restructuring. This is observed in the side of club 2, for example, where large structures are present with X-ray absorbance similar to that of the impact region, suggesting a much higher local degree of mineralization, surrounded by regions of low X-ray absorbance, presumably regions without material. Virtual 3D renderings of clubs 1 and 2 with a cut-out from behind reveal that this structure – when present – stretches throughout the club and follows the club curvature [Fig. 1 ▸(*e*) and 1(*f*)]. Even when this morphological deviation is not present, the densities of both the periodic and striated regions in the side of the club differ from that of the periodic region close to the impact region [Figs. 1 ▸(*c*) and 1 ▸(*e*)]. Although large restructuring is clear in club 2, clubs 3–5 show no such restructuring, as shown in Fig. 2 ▸.

Lab-µCT investigations of the entire bodies of animals 3–5 are presented as renderings in Figs. 2 ▸(*a*)–2(*f*), which clearly illustrate that the dactyl club is the densest material in the stomatopod cuticle. Higher-resolution lab-µCT investigations of extracted and epoxy-embedded clubs were carried out [Figs. 2 ▸(*g*)–2(*l*)]. From the renderings of the whole clubs, it is clear that the impact surface and part of the impact region (Chua *et al.*, 2023 ▸; Huang *et al.*, 2020 ▸) have been partially worn off all the clubs from use-induced wear [Figs. 2 ▸(*g*)–2(*i*)]. Much like club 1 [Fig. 1 ▸(*c*)], all the slices through these clubs show a higher density in the side of the club than in the periodic region close to the impact region [Figs. 2 ▸(*j*)–2 ▸(*l*)]. The lab-µCT scans of the dactyl clubs were made with a 4.5 µm voxel size. Surprisingly, even at this resolution, virtual slices through the clubs revealed no calcite-band-like morphology present in any samples [Figs. 2 ▸(*j*)–2 ▸(*l*)]. Thus, it seems that the large restructuring observed in club 2 is uncommon, as only one of the five clubs scanned with a sub-5 µm resolution has shown this.

### 3.2. Synchrotron XRD/XRF experiments

**Figure 3:**
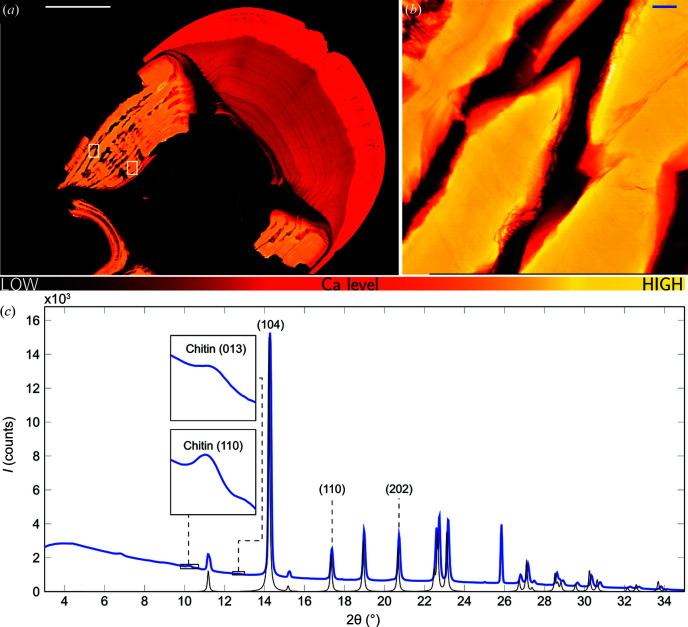
2D scanning XRD/XRF of a slice from club 6. (*a*) Overview Ca *K* XRF map (scale bar 1 mm, colour gradient bar shows the Ca content). White boxes mark region 1 (left) and region 2 (right) scanned at higher in-plane resolution. (*b*) High-resolution Ca *K* XRF map of region 1 (scale bar 20 µm). (*c*) Mean diffraction pattern of region 1 (blue) with a model diffractogram of calcite (black). Insets show the chitin diffraction peaks. The peaks marked by (*hkl*) were used for further analysis of the orientation and spatial distribution of calcite [reflections (104), (110) and (202)] and chitin [reflections (110) and (013)].

To obtain detailed structural information of the regions with the large restructuring observed in the sides of club 2 (Fig. 1 ▸), a slice extracted from club 6, which has a similar restructuring to club 2, was investigated by synchrotron sub-micrometre scanning XRF/XRD (Fig. 3 ▸). The XRF maps of the altered microstructure in the striated and periodic regions at the side of the club have a higher Ca content than in the impact region [Fig. 3 ▸(*a*)]. This higher Ca content in the morphological altered region with respect to the impact region indicates that the modified side region must be very highly mineralized locally. We note that impact studies have shown the impact region to be among the strongest biological materials (Stegbauer *et al.*, 2021 ▸; Marcus *et al.*, 2017 ▸; Amini *et al.*, 2015 ▸; Weaver *et al.*, 2012 ▸). Scanning a region of this side structure of the club with a higher resolution showed that the Ca content in the side of the club is highly spatially heterogeneous with bands of highly concentrated Ca regions interspersed by regions with virtually no Ca [Fig. 2 ▸(*b*)]. This is in agreement with the observations using tomography on club 2 (Fig. 1 ▸).

The mean diffractogram of the high-resolution map of region 1 of club 6 [Fig. 3 ▸(*b*)] matches that of calcite with a minor signal from chitin [Fig. 3 ▸(*c*)]. This shows that the modified side region contains calcite crystallites and, comparatively weakly scattering chitin. No other crystalline phases were observed and there was no signal corresponding to ACC or ACP as observed in the periodic region close to the impact region (data not shown). Calcite and chitin are common in cuticles of crustaceans, such as shrimp, lobster and blue crabs (Nekvapil *et al.*, 2020 ▸; Gbenebor *et al.*, 2017 ▸; Kunkel *et al.*, 2012 ▸). However, calcite has not previously been reported to occur in stomatopod dactyl clubs. Instead, they have been described as consisting mainly of chitin, Ap, ACC and ACP (Amini *et al.*, 2019 ▸; Weaver *et al.*, 2012 ▸).

**Figure 4:**
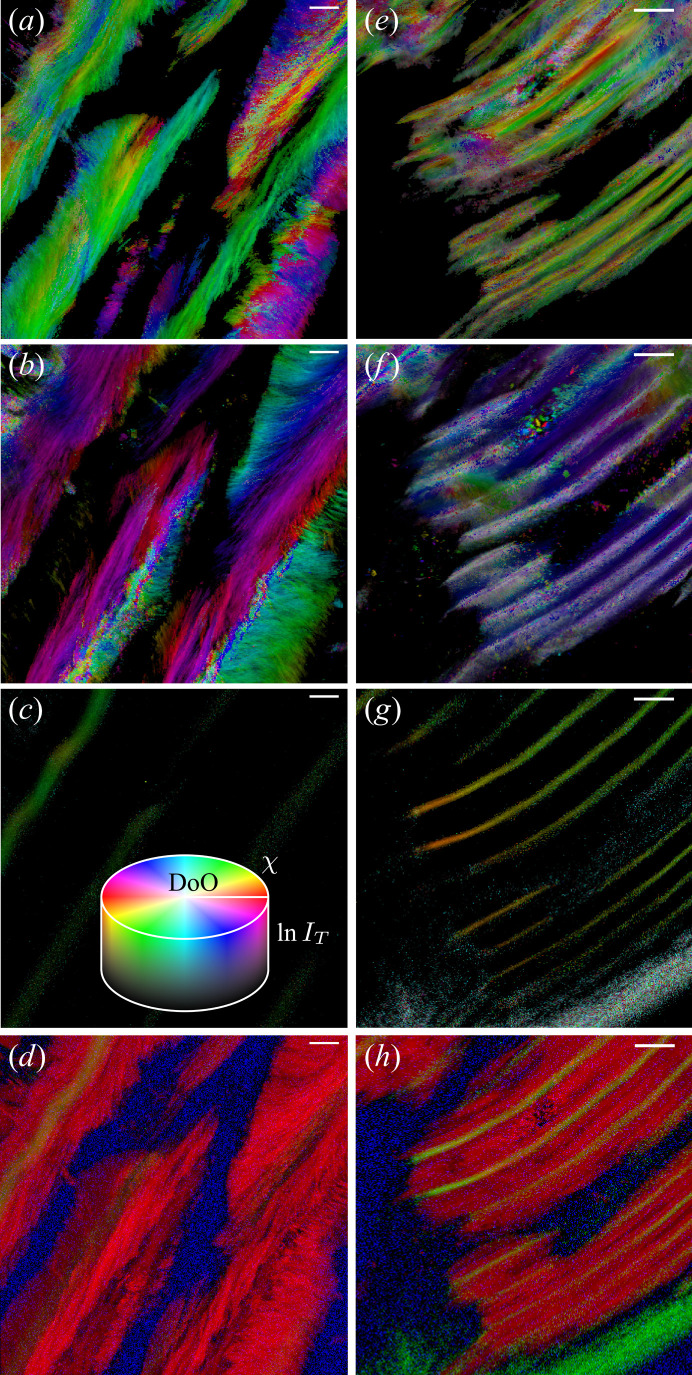
Distribution and orientation of calcite and chitin in the side of club 6. (*a*)–(*d*) Region 1. (*e*)–(*h*) Region 2. (*a*), (*e*) Orientation of calcite (104). (*b*), (*f*) Orientation of calcite (110). (*c*), (*g*) Orientation of chitin (013). The inset in (*c*) shows how to interpret the figures: hue – orientation, saturation – projected degree of orientation (DoO), and value – natural logarithm to the total peak intensity in each point. (*d*), (*h*) RGB correlation of calcite and chitin: red – calcite (104) and (110), green – chitin (013), blue – chitin (110). The scale bars are 20 µm.

The results of the orientation analysis performed on the high-resolution regions are shown in Fig. 4 ▸, where orientation, projected degree of orientation and diffraction intensity are given by colour, saturation and value in each pixel, respectively. The projected degree of orientation is the degree of orientation visible in the projection, *i.e.* the orientation projected onto the detector. Two different structural motifs are present in the scanned regions: in region 1 of club 6, the calcite crystallites are arranged in sparse large feather-like structures [Figs. 4 ▸(*a*)–4(*d*)]. In region 2 of club 6, a dense set of calcite bands are present [Figs. 4 ▸(*e*)–4(*h*)]. In the centre of both the calcite feathers and the bands, there is a high in-plane (013) orientation of chitin, which is oriented along the long direction of the calcite crystallite morphology [Figs. 4 ▸ (*c*)–4(*g*)]. Here, it is also seen that the distance between oriented chitin bands is smaller in region 2 than region 1 of club 6. In the feathers, the calcite (110) orientation radiates out from the central oriented chitin line [Fig. 4 ▸(*b*)]. Similar trends are observed, albeit to a lesser degree, in the band structures [Fig. 4 ▸(*f*)]. Comparison of the calcite (104) and (110) orientation maps [Figs. 4 ▸(*a*), 4(*b*), 4(*e*) and 4(*f*)] shows that the calcite crystallites are semi-continuously oriented and follow the larger scale structure of the feathers/bands. This suggests that the calcite crystal forms by templating of the oriented chitin component; see the overlap maps of chitin and calcite diffraction signals in Figs. 4 ▸(*d*) and 4 ▸(*h*). From these maps, it is clear that the unoriented chitin (110) peak is not colocalized with the highly oriented (013) chitin peak, suggesting the presence of two chitin components.

A similar experiment was carried out on club 7, which was harvested 7 days after moulting and investigated by overview maps. A correlation map of Ap, Ca XRF, and calcite (104) and (202) is shown in Fig. S1 of the supporting information, from which it is clear that calcite forms near the back of the club farthest from the impact surface. This shows that the calcite can form very early in the mineralization process of the club. Thus, the presence of calcite does not seem to constitute a significant hindrance for the life of the stomatopods. The majority of club 7 consists of ACC and ACP, and the striated and periodic region in the sides of the club shows no sign of calcite crystallites, which are only present in the back of the club.

In order to determine whether the seemingly unmodified, and thus presumably fully amorphous, clubs of animals 3–5 indeed do not present crystalline calcite, thin slices of clubs 3–5 were investigated by position-resolved XRD. For club 3, no calcite was detected, which is consistent with the lack of microstructural alterations observed by µCT. The set of diffractograms can be split into a subset with a large amount of Ap and a subset with a large amount of chitin. These distribute into two spatially distinct areas in the club.

**Figure 5:**
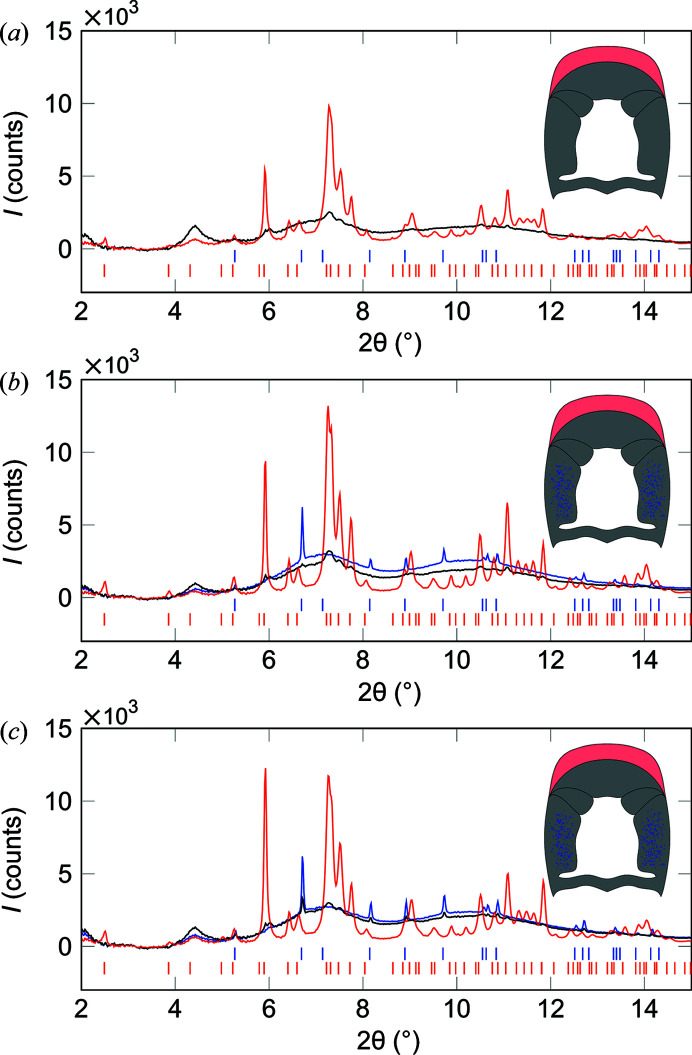
Diffractograms from clubs (*a*) 3, (*b*) 4 (*c*) and 5. Red diffractograms stem from areas with high Ap (002) content, black diffractograms from areas with high chitin (110) content, and blue diffractograms show areas with high calcite (104) and (202) content. Blue tick marks indicate standard calcite (Maslen *et al.*, 1995 ▸), red tick marks indicate standard Ap (Wilson *et al.*, 1999 ▸). The sketches in each panel show the approximate localization of the phases within the clubs. In (*a*) there is no blue diffractogram or blue dots in the as no calcite was found in club 3.

The mean diffractograms for these areas show that Ap was localized in the impact region, while the amorphous scattering and chitin diffraction signals stem from the periodic and side regions of the club as sketched [Fig. 5 ▸(*a*)]. The diffractograms of clubs 4 and 5 contain small localized regions displaying calcite diffraction patterns. The average diffractograms of areas containing calcite, Ap and chitin show that Ap is localized in the impact region, chitin and amorphous phases in the periodic and striated region, while calcite-containing areas are localized to the side of clubs 4 and 5, as indicated by the sketches in Figs. 5 ▸(*b*) and 5 ▸(*d*). In contrast to the large restructuring in clubs 2, 6 and 7 [Figs. 1 ▸(*d*), 3 ▸(*c*) and S1(*b*)], the calcite peaks in clubs 4 and 5 are small compared with the amorphous background.

**Figure 6:**
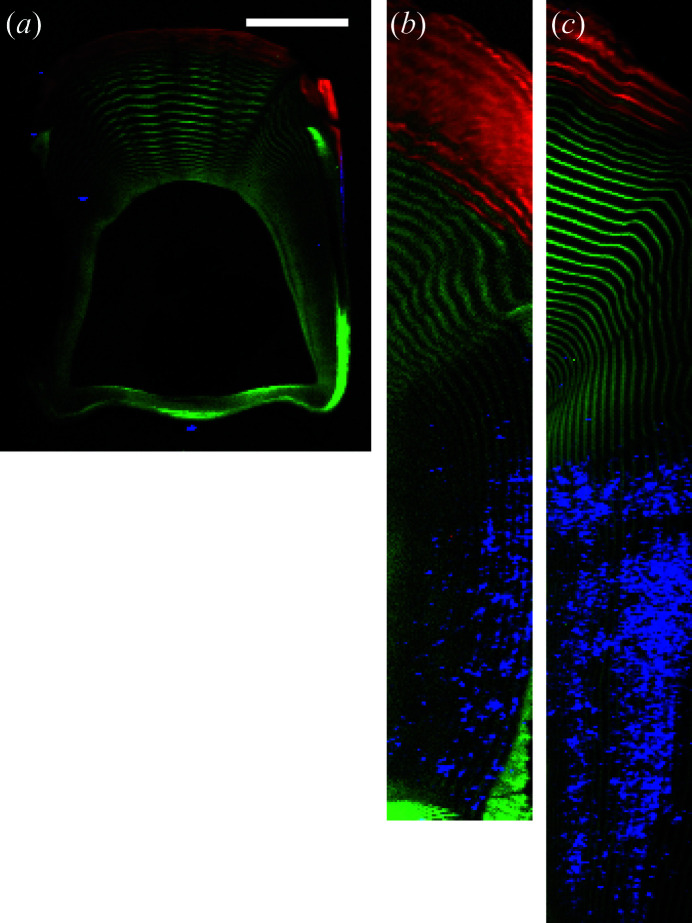
Correlation maps in RGB for clubs (*a*) 3, (*b*) 4 and (*c*) 5 with: red – Ap (002), green – chitin (110), and blue – calcite (104) and (202). Note that no calcite is present in (*a*); inspection of the diffractograms revealed that the blue dots are contaminants with diffraction peaks that coincide with either the (104) or the (202) calcite peaks. The scale bar in (*a*) is 500 µm and all figures were scaled accordingly. The intensity scale of the blue and green channels does not represent the full signal scale since isolated regions with orientation-induced very intense signals would otherwise make the chitin and calcite structures near-invisible.

Since the voxel size of the µCT used to image these clubs was 4.5 µm, and calcite crystallites were not identified morphologically therein, the crystals in the clubs must be smaller than ∼9 µm. The beam used in the XRD experiment was 20 × 10 µm with a 100 µm sample thickness. Given the relatively small calcite diffraction signals compared with the larger amorphous peaks in the blue diffractograms [Figs. 5 ▸(*b*) and 5(*c*)], the transformation from amorphous phase to calcite thus seems incomplete in comparison with the seemingly full transformation in clubs 1 and 7. The majority of the sides of clubs 4 and 5 are most likely still weakly scattering ACC and ACP, as evident from the relatively high intensity of the amorphous peaks compared with the calcite peaks (Fig. 5 ▸). The sketches in Fig. 5 ▸ summarize the material distribution: Ap in the impact region, chitin in the periodic and striated regions, and small calcite particles in the side of clubs 4 and 5, but absent in club 3. Composite maps of the different phases based on the intensity of selected diffraction peaks can show the localization of the different phases (Fig. 6 ▸). As there is Ap present in these scans, unlike in the scan of region 1 in club 6 (Fig. 4 ▸), only the chitin (110) is used to map the chitin distribution, as the Ap (002) and chitin (013) peaks overlap. The maps show Ap (002) in red, chitin (110) in green, with blue showing a mix of calcite (104) and (202) (Fig. 6 ▸). In club 3, only Ap and chitin are present [Fig. 6 ▸(*a*)]. A few minor blue points are observed that result from an unidentified contaminant, with diffraction peaks coinciding with either calcite (104) or calcite (202), and do not reflect calcite. For clubs 4 [Fig. 6 ▸(*b*)] and 5 [Fig. 6 ▸(*c*)], there is a clear distribution of calcite particles throughout the sides of the clubs. Some of the regions cover hundreds of micrometres. The fact that this microstructure was not spatially resolved by µCT suggests that these regions consist of multiple smaller crystallites.

The fact that calcite crystallites were detected in the XRD experiment but not in the lab-µCT experiment might suggest that the sample preparation or sample drying caused nucleation of calcite crystallites. To examine this possibility, the slice of club 3 was heated to 373 K in a vacuum oven for 2.5 h and re-examined by position-resolved XRD. The diffractograms were then spatially separated in the same way as in Figs. 4 ▸ and 5 ▸. No calcite had formed in the side regions of the club [Fig. S2(*a*)]. The required sample manipulation resulted in the sample breaking into several larger pieces and Fig. S2(*a*) thus only shows a single example of an intact region. However, the remainder of the club did not show signs of calcite formation either. The mean diffractogram of the Ap-rich and chitin-rich areas did not reveal any calcite either [Fig. S2(*b*)]. Thus, no crystallization occurred even after 2.5 h of vacuum treatment at 373 K that should have induced dehydration. Hence, the biogenic ACC and ACP in the club are quite stable. Thus, it is unlikely that the sample preparation process affected the structure.

The observations made on the examined clubs are summarized in Table 1 ▸. Taken together, they show that there must be a degree of flexibility in the design of the stomatopod dactyl club. In the sides of the clubs from multiple specimens, we found examples of massive calcite structures, small calcite crystallites interspersed in the amorphous mineral or no calcite at all. When only amorphous phases were present in the side of the club, they were stable to drying and abrasion. There are in fact plenty of examples of stable ACC and ACP biominerals, including chiton teeth, lobster cuticle and Ca storage organs in crayfish (Stegbauer *et al.*, 2021 ▸; Fabritius *et al.*, 2009 ▸; Luquet & Marin, 2004 ▸), so it is possible that the crystallization of calcite crystallites is by design.

The design flexibility in the side regions of the club, described in the present study, suggests that the mechanical challenges the club is subjected to can propagate through the various structural elements. In turn, we speculate that the presence of chitin sheets partially stiffened by either crystalline or amorphous mineral may suffice to withstand mechanical load. However, we cannot exclude the possibility that the crystallization of the amorphous phase in the sides of the club into calcite reflects ageing of the club. Although calcite crystals were also observed in club 7 only 1 week after moulting, club age cannot be fully excluded as a factor for the process. Therefore, an alternative explanation could be that the amorphous phase in the sides of the club is only sufficiently stabilized to be structurally sound through the typical time between moulting until the new club is complete. During moulting, a completely new club structure is formed, whereby the amorphous sides are renewed (Chua *et al.*, 2023 ▸; Amini *et al.*, 2019 ▸). Thus, this may reflect that evolution drives towards solutions that are ‘good enough’ rather than optimal.

## 4. Conclusions

We have shown that calcite sometimes forms in the side region of the stomatopod dactyl club in the species *Odontodactylus scyllarus*. These structures can form as early as 1 week after moulting, when a new club has started to grow. The morphology and distribution of the calcite crystallites can vary drastically from club to club, and the amorphous phase, when the calcite crystallites are not present, is very stable. When the calcite crystallites grow larger, they seem to organize around the chitin bands found in the periodic and striated regions of the dactyl club. The results suggest that the clubs can accept a certain degree of design flexibility.

## Supplementary Material

Supporting figures. DOI: 10.1107/S2052252523002075/fc5066sup1.pdf

## Figures and Tables

**Table 1:** Overview of the experiments performed on the various samples involved in this work

Club	Experiments	Lab-µCT	Synchrotron	Origin	Animal length (cm)
1	Lab-µCT	No restructuring	–	Singapore	
2	Lab-µCT	Large restructuring	–	Singapore	
3	Lab-µCT, scanning XRD	No restructuring	No calcite	Stockholm	11
4	Lab-µCT, scanning XRD	No restructuring	Calcite	Stockholm	13
5	Lab-µCT, scanning XRD	No restructuring	Calcite	Stockholm	16
6	Scanning XRD and XRF	Large restructuring	Calcite	Singapore	
7	Scanning XRD and XRF	–	Some restructuring, calcite identified by XRD	Singapore	
