# Supplementary material for: Flexible design in the stomatopod dactyl club
Source: IUCrJ. 2023 Mar 14;10(Pt 3):288–96. doi: 10.1107/S2052252523002075 (PMC10161772; doi:10.1107/S2052252523002075)
Supplement: Supplementary file 1 [file m-10-00288-sup1.pdf]

IUCrJ

Volume 10 (2023)

Supporting information for article:

Flexible design in the stomatopod dactyl club

Thorbjørn Erik Køppen Christensen, Jia Qing Isaiah Chua, Nina Kølln Wittig, Mads Ry Vogel Jørgensen, Innokenty Kantor, Jesper Skovhus Thomsen, Ali Miserez and Henrik Birkedal

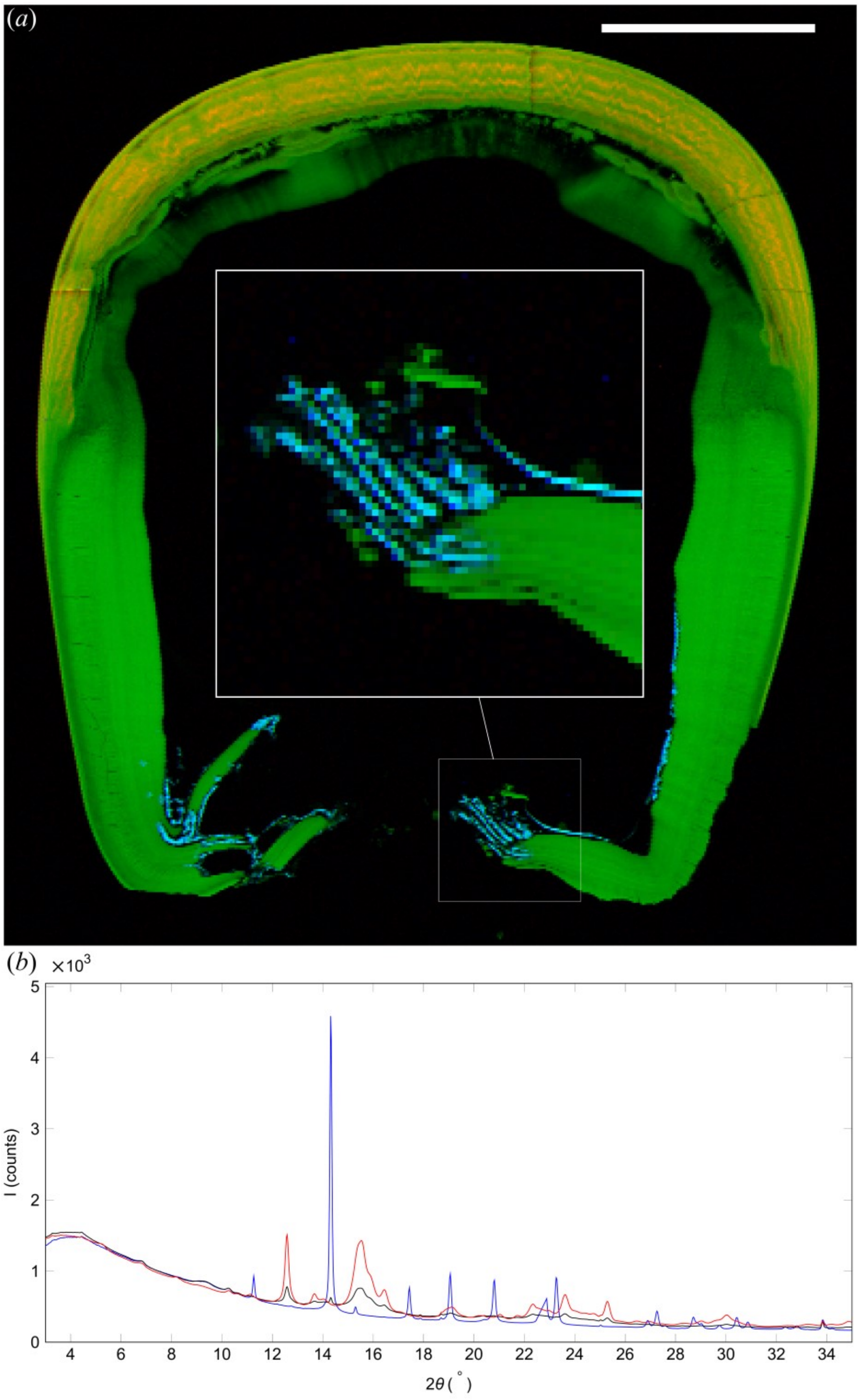

Figure S1 XRD and XRF scan of club 7. (*a*) RGB correlation map, red = Ap (002), green = Ca XRF, blue = calcite (104) and (202). Scale bar 1 mm. The inset shows an area with high calcite content magnified by a factor of three. (*b*) Average diffraction pattern of areas with high Ap (002) (red), chitin (110) (black), and calcite (104) and (202) (blue).

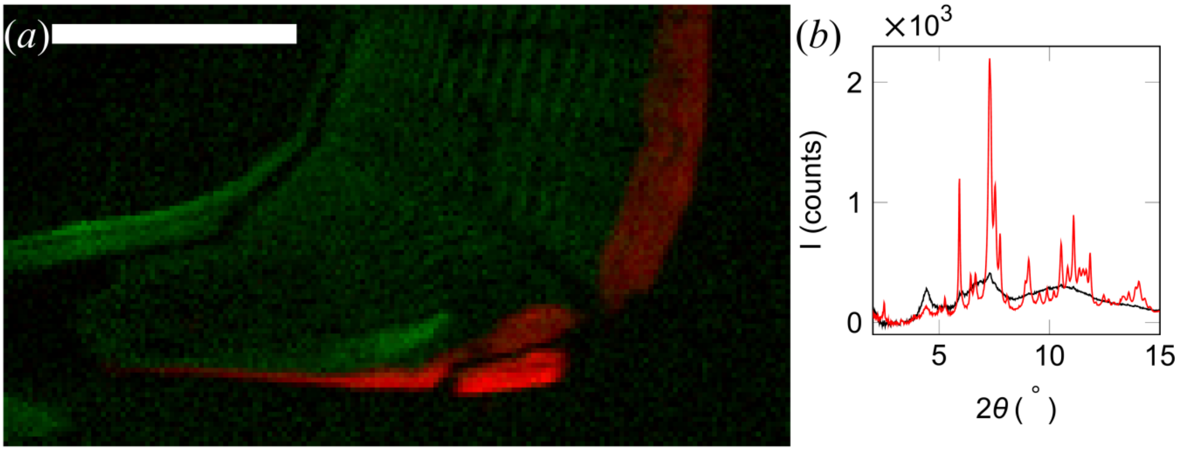

Figure S2 Club 3 after heating in vacuum oven at 373 K for 2 h 30 min. (*a*) RGB correlation image with red = Ap (002), green = chitin (110), blue = calcite (104) and (202). Scale bar 500 µm. (*b*) Diffraction patterns of the area with high Ap intensity (red, red area in (*a*)) and high chitin intensity (black, green area in (*a*)) in (*a*).
